# Editorial: Motivations for physical activity—volume IV

**DOI:** 10.3389/fpsyg.2026.1952408

**Published:** 2026-08-19

**Authors:** Iuliia Pavlova, Pedro Morouço, Aleksandra M. Rogowska

**Affiliations:** 1 Ivan Boberskyj Lviv State University of Physical Culture, Lviv, Ukraine; 2 University of Helsinki, Helsinki, Finland; 3 National Program for the Promotion of Physical Activity, Directorate-General of Health, Lisbon, Portugal; 4 Institute of Psychology, University of Opole, Opole, Poland

**Keywords:** behavior change, capability, maintenance, motivation, physical activity, self-efficacy, sport psychology

Physical activity promotion is often conceptualized and studied as if starting and staying are the same problem. The 29 contributions to this Research Topic suggest that they are not. Across school, university, workplace, community, sport, rehabilitation, and clinical settings, the authors show that the factors influencing participation in physical activity are not the same at different points during engagement with a behavior change program, and that support which may be adequate or even critical at one juncture can be detrimental at another. This conclusion has clear implications for practice, since if the determinants of initiation and maintenance differ, as the present issue demonstrates, then interventions need to be differentiated according to the stage of change of the target population. The volume gathers original survey research, qualitative studies, narrative and systematic reviews, a theoretical analysis, a clinical pilot, and a randomized controlled trial, conducted across different cultural and policy settings. Together, these studies show how motivation can lead to action and continued participation, and where this process may fail.

## Initiating and maintaining physical activity are different processes

The clear indication of this asymmetry comes from a test of the Multi-Theory Model among patients receiving maintenance hemodialysis. Intention to engage was predicted by behavioral confidence, perceived advantages and disadvantages, and changes in the physical environment. Intention to maintain it was predicted by emotional transformation, practice for change, changes in the social environment (Zhang et al.). Beginning depends on whether physical activity is perceived as feasible and worthwhile. Continuing depends on coping, self-regulation, and support that outlasts the initial decision. Since both variables measure the intention to engage in behavior at a particular moment, it is more appropriate to regard this data as an indication of a possible theoretical framework in which these constructs require further longitudinal investigation rather than a sequence of stages.

## Beginning: value, confidence, and a feasible opportunity

Expectancy-value theory deals primarily with content: what has to be there at the beginning. It focuses on two questions: “Can I do this?” and “Why is it worth doing?” The first refers to confidence in one's ability, while the second includes enjoyment, usefulness, personal meaning, and cost. Meta-analytic evidence confirms both constructs as related to engagement, persistence, intention, and performance, although nearly all the studies were cross-sectional in design (Song and Choi). Cost deserves particular attention, since it reaches well beyond physical effort into time, anxiety, expense, discomfort, missed alternatives, and anticipated failure. Each of the two aspects is present in the Research Topic. On the value side, the intention to run a marathon was associated with not only internal motives but also the perception of challenges, symbolic and social value, cultural background, and practical convenience, as well as self-efficacy partially carried those perceptions into intention (Wang M. et al.). In terms of environment, biophilic elements in sports facilities, such as natural light, vegetation, views, and natural materials, were associated with exercise immersion and with the intention to continue (Lee and Park). A setting does not merely permit movement; it shapes how movement feels. Where activity carries symptoms or clinical risk, confidence appears to be a critical factor. Among hemodialysis patients, behavioral confidence was the strongest predictor of intention to start, ahead of perceived advantages and disadvantages and of changes in the physical environment (Zhang et al.). The finding highlights this group's lack of awareness of the connection between activity and their health: health information provides an external source of knowledge about the benefits but not about the risks and opportunities.

## The distance between intention and action

An intention formed is not yet a behavior performed, and a few articles highlight processes related to both stages. Among university students, exercise self-efficacy was associated with participation through greater interest in sport learning and lower procrastination (Yong and Fan). Believing that one can exercise may support participation, but people still need to overcome delays, poor time management, and competing priorities. Physical activity competes with various engaging and addictive behaviors that have become an integral part of contemporary lifestyles. Exercise adherence was associated with reduced mobile-phone addiction, probably through decreased boredom and increased self-control processes (Qi et al.). At this stage, practical support is especially important. For children, services such as transportation, registration, payment, and access to resources were more strongly associated with moderate and moderate-to-vigorous physical activity than encouragement or parent involvement. However, different types of assistance were linked to varying activity levels, and the results should not be generalized beyond parental support (Wang S. et al.). The same applies to the mutual influence of adults and children, where sport promotion was related to both parental and children's motivation (Yang et al.).

Digital tools can promote intention–action consistency and adherence. Online fitness content was associated with better planning and more positive feelings, which were in turn linked to greater participation in resistance training. However, these associations appeared to weaken with longer daily exposure, including 30 mins or more per day (Wei et al.). A Strava-supported blended physical education model was valued for personalization, feedback, and its non-competitive structure, yet many students completed only the minimum required distance or time (Hrušová et al.). Engagement with an app is not equivalent to autonomous behavior change. A conceptual analysis of two health projects argues that such technologies should support reflection, value alignment, and internalization instead of maximizing app engagement, so that users grow less dependent on prompts (Gerstenberg et al.). Design intent, however, does not guarantee results. A randomized controlled trial among Finnish military employees, combining a smartphone-linked accelerometer with feedback, goals, brief counseling, and encouragement to exercise during working hours, did not produce detectable between-group change in physical activity, fitness, body composition, cardiometabolic biomarkers, stress, or work ability (Pietiläinen et al.). It is a caution against assuming that self-monitoring translates into change.

## Staying: commitment, negotiation, and adaptation

Once activity has begun, different mechanisms take over, and motivation itself recedes as the proximal driver. Sport commitment was positively associated with the intention to continue exercising among Chinese CrossFit practitioners, mediating the relationship between motivation and continuation, while community affiliation had no effect, direct or indirect (Ji and Wu). Two cautions apply: the outcome of interest was the intention rather than behavior, and the sample included only current exercisers who presumably had decided to continue.

Self-Determination Theory addresses the phenomenon of commitment theoretically: while this review article focuses on sport commitment, the mechanisms appear to be applicable to other settings too. A narrative review of sport motivation gathers the relevant argument for athletes, foregrounding enjoyment, self-determination, coaching climate, and personal goals, while noting that external rewards and recognition can still support participation provided they do not crowd out autonomy (Alkasasbeh and Akroush). Structures recruiting people through requirement or incentive would need to convert compliance into something self-endorsed to hold them once the incentive ends. Negotiation is another process that emerges when analyzing the barriers to participation. Among the participants in ice and snow sports, leisure motivation was unrelated to recreation specialization, while the indirect relationship was revealed through the use of negotiation tactics to manage access, opportunity, affordability, skill, and equipment (Liang et al.). The intention to continue is not sufficient; people also need the skills and strategies to overcome barriers.

## What conditions participation at every stage

Some factors do not belong to one stage but bear on all of them. Capability is the prime example, and its role emerges as both preceding and following behavior. Capability informs general feelings of perceived ability from the start but is also subject to development, rehabilitation, injury, aging, and other influences. For this reason, the COM-B model conceptualizes capability, along with opportunity and motivation, as a variable that affects the other two rather than a linear sequence. A study on older people identified motivation, social and environmental supports, and functional capacity as highly intertwined concepts, with social and environmental supports being associated with activity involvement partially through motivation and with functional capacity associated with these connections (Wei and Wang). Fatigue, pain, limited functionality, restrictions to movement, the fear of falls, and the absence of adapted opportunities undermine confidence, increased cost-benefit evaluation, and perception of value before and after engagement. Opportunities, in turn, can be more pervasive and multifaceted. A cardiac rehab pilot study identified three preliminary profiles of patients with varying patterns of contributing factors—reduced physical capacity as a limiting factor, combined motivational and environmental barriers, and organizational barriers (Molina et al.). Similarly, wheelchair curling athletes described a similar pattern: enjoyment, competence, belonging, ambition, and identity supported participation, while inaccessible facilities, unsuitable equipment, transport problems, and limited organizational support made it harder to continue (Daşkesen et al.). Motivation alone is not enough when the system is inaccessible.

Cultural and environmental context sets further limits, at least as participants experience it. Adults in Saudi Arabia who participated in outdoor sport for health, mood, sociability, and enjoyment talked of family responsibilities, costs, boredom, lack of suitable facilities and safety, gender norms, and the absence of supportive policies and media (Albujulaya and Stevinson). These are accounts of constraint rather than an objective comparison of policy environments. Motivation and inactivity can coexist. Promotion must therefore also remove reasons to avoid, not only supply reasons to participate. Weight stigma was associated with exercise avoidance both directly and indirectly through internalized stigma and social inhibition with potential implications for sport settings that could make individuals with negative body image feel unwelcome (Li Q. et al.). Interventions should therefore avoid appearance-based pressure and negative messages about weight. Exercise avoidance is not simply a lack of motivation; it is an active response that requires different forms of support.

## Meanings and returns beyond participation

Several contributions explore the links between the initial motivation and participation and related psychological benefits, conceptualized in terms of identity, values, beliefs, prosocial behaviors, and positive psychology. These are valuable additions to the literature on social cognitive mechanisms in physical activity as all of these factors can act as reinforcers of sustained engagement, although the review does not include evidence on subsequent participation.

Participation is also connected to how people see themselves. Students who perceived stronger involvement from school leaders reported more autonomous motivation, regardless of whether they were currently active or how long they had been in the program (Guimaraes et al.). Among high school athletes, different aspects of self-concept—academic, family, social, emotional, and physical—were related in different ways to motivation and the needs for autonomy, competence, and relatedness. This suggests that motivation is linked to the whole sense of self, not only to sporting ability (Jakobsen). Pole dance was associated with small but consistent improvements in self-esteem, global interoceptive awareness, and reduced interoceptive worry with no differences in self-efficacy beyond activity-specific self-confidence (Rogowska et al.).

Activity also relates to outcomes in other domains. Physical activity was associated with life satisfaction through resilience, with mindfulness moderating the pathway, among sports science students (Secer et al.). Exercise involvement was associated with career decision-making self-efficacy beliefs through similar mechanisms of increased self-awareness and resilience, potentially linking sport and general work engagement (Niu et al.). Jiang et al. position exercise as a potential resource for stress management and resilience in neurodegenerative disease.

The meanings derived from participation may also go beyond intrinsic and extrinsic motivation. Individual and team outdoor athletes differed in their explicit, but not implicit, willingness to endorse unsustainable options and rejected the concept of sustainable development in similar ways, reflecting values that are accessible to awareness as well as universally desired (Hoja and Jansen). Egoistic motives of skill development, recognition, and networking were associated with sports volunteering, where social norms and organizational support were key factors, suggesting that self-motivated behavior can have genuinely prosocial outcomes (Zhang et al.). In soccer players, hindsight bias was modulated by win-loss outcome, playing status, and related to the judgment of responsibility, control, and predictability, with players in winning teams reporting the highest levels of bias (Li T. et al.).

## Strength of the evidence

The designs assembled here differ in strength. The meta-analysis offers the widest synthesis, though 29 of its 30 studies were cross-sectional and heterogeneity was substantial, so it establishes the breadth and consistency of expectancy-value associations rather than their causal direction. The randomized controlled trial detected no between-group change; with 47% attrition and reduced power for small effects at follow-up, this is best read as a failure to detect benefit rather than as evidence that no benefit exists. Qualitative studies provide contextual insight into how barriers are experienced rather than estimates of how common they are. Cross-sectional mediation models, which form the largest share of the volume, identify plausible mechanisms that require temporal confirmation, and several measure intention rather than observed behavior. The clustering pilot in cardiac rehabilitation is exploratory.

The Research Topic also spans children, adolescents, students, older adults, recreational and elite athletes, volunteers, military employees, and clinical and disabled populations, across settings including China, South Korea, Saudi Arabia, Finland, Canada, Türkiye, Norway, Poland, Germany, France, and Jordan. That range reinforces one of its own conclusions, namely that because motivation is situated, findings and interventions do not transfer unchanged across cultures, populations, or settings. Taken as a whole, the volume maps the territory of motivation convincingly and establishes causal relationships in very little of it.

## Future perspectives: strengthening the evidence

Calling for more longitudinal and experimental research is not enough; future studies should address specific gaps. The contributions here point to more specific priorities. Studies should distinguish initiation, maintenance, lapse, and re-entry rather than treating participation as a single state, and should measure observed behavior, with objective monitoring where feasible, instead of relying on intention. They should recruit people who have discontinued alongside current participants, since samples of active people cannot explain dropout. They should test whether proposed mediators change before behavior changes, which is the minimum condition for a mechanistic claim. They should compare stage-matched interventions against generic ones, examine whether the same mechanism operates across cultural and clinical contexts, and evaluate the implementation burden, acceptability, and unintended effects of digital tools rather than their engagement metrics alone.

## Conclusion

The studies in this volume support one practical message: physical activity promotion should match the person's stage of participation. Starting activity requires perceived value, confidence, interest, and a realistic opportunity to take part. Continuing requires commitment, strategies to overcome barriers, adaptation to changing abilities, and ongoing support. At both stages, participation is also shaped by capability, environment, culture, and stigma. The goal is therefore not merely to increase motivation, but to provide the right support when participation is most at risk.

